# Magnetic Properties of Bacterial Magnetosomes Produced by *Magnetospirillum caucaseum* SO-1

**DOI:** 10.3390/microorganisms9091854

**Published:** 2021-08-31

**Authors:** Kamil G. Gareev, Denis S. Grouzdev, Peter V. Kharitonskii, Demid A. Kirilenko, Andrei Kosterov, Veronika V. Koziaeva, Vladimir S. Levitskii, Gabriele Multhoff, Elina K. Nepomnyashchaya, Andrey V. Nikitin, Anastasia Nikitina, Elena S. Sergienko, Stanislav M. Sukharzhevskii, Evgeniy I. Terukov, Valentina V. Trushlyakova, Maxim Shevtsov

**Affiliations:** 1 Department of Micro and Nanoelectronics, Saint Petersburg Electrotechnical University “LETI”, 197376 Saint Petersburg, Russia; nikitinandrew2@gmail.com (A.V.N.); e.terukov@hevelsolar.com (E.I.T.); vvtrushliakova@mail.ru (V.V.T.); 2 SciBear OU, Tartu mnt 67/1-13b, Kesklinna Linnaosa, 10115 Tallinn, Estonia; denisgrouzdev@gmail.com; 3 Department of Physics, Saint Petersburg Electrotechnical University “LETI”, 197376 Saint Petersburg, Russia; peterkh@yandex.ru (P.V.K.); nastya_nikitina1996@mail.ru (A.N.); 4 Centre of Nanoheterostructure Physics, Ioffe Institute, 194021 Saint Petersburg, Russia; zumsisai@gmail.com; 5 Department of Earth Physics, Saint Petersburg University, 199034 Saint Petersburg, Russia; a.kosterov@spbu.ru (A.K.); e.sergienko@spbu.ru (E.S.S.); 6 Research Center of Biotechnology of the Russian Academy of Sciences, Institute of Bioengineering, 119071 Moscow, Russia; vkoziaieva@mail.ru; 7 R&D Center TFTE LLC, 194021 Saint Petersburg, Russia; v.levitskiy@hevelsolar.com; 8 Center of Translational Cancer Research (TranslaTUM), Klinikum Rechts der Isar, Technical University Munich, 81675 Munich, Germany; gabriele.multhoff@tum.de (G.M.); shevtsov-max@mail.ru (M.S.); 9 Institute of Electronics and Telecommunications, Peter the Great St. Petersburg Polytechnic University, 195251 Saint Petersburg, Russia; elina.nep@gmail.com; 10 Magnetic Resonance Research Centre, Saint Petersburg University, 199034 Saint Petersburg, Russia; stanislav.sukharzhevskii@spbu.ru; 11 Laboratory of Biomedical Nanotechnologies, Institute of Cytology of the Russian Academy of Sciences, 194064 Saint Petersburg, Russia; 12 Personalized Medicine Centre, Almazov National Medical Research Centre, 197341 Saint Petersburg, Russia

**Keywords:** magnetotactic bacteria, *Magnetospirillum caucaseum* SO-1, bacterial magnetosomes, magnetostatic interaction, theoretical modeling, magnetic properties

## Abstract

In this study, the magnetic properties of magnetosomes isolated from lyophilized magnetotactic bacteria *Magnetospirillum caucaseum* SO-1 were assessed for the first time. The shape and size of magnetosomes and cell fragments were studied by electron microscopy and dynamic light scattering techniques. Phase and elemental composition were analyzed by X-ray and electron diffraction and Raman spectroscopy. Magnetic properties were studied using vibrating sample magnetometry and electron paramagnetic resonance spectroscopy. Theoretical analysis of the magnetic properties was carried out using the model of clusters of magnetostatically interacting two-phase particles and a modified method of moments for a system of dipole–dipole-interacting uniaxial particles. Magnetic properties were controlled mostly by random aggregates of magnetosomes, with a minor contribution from preserved magnetosome chains. Results confirmed the high chemical stability and homogeneity of bacterial magnetosomes in comparison to synthetic iron oxide magnetic nanoparticles.

## 1. Introduction

Because of its unique magnetic properties and due to a large number of organisms across biological domains capable of producing it, biogenic magnetite has attracted a constant interest since its discovery [1,2,3,4]. Due to its wide distribution in nature, one of the most studied magnetite producers is magnetotactic bacteria (MTB) biomineralizing nanosized to submicron single crystals of magnetite in special organelles, referred to as magnetosomes that are responsible for MTB magnetoreception [3]. During the subsequent fossilization of dead MTB, magnetofossils are formed carrying paleomagnetic signal in a variety of sedimentary environments, from brackish and freshwater [5,6,7,8] to marine [9,10,11]. Stable remanent magnetization of magnetofossils is carried by single-domain [12,13] and exceptionally [14] pseudo-single-domain magnetite, which, due to the chemical composition and shape of individual magnetite crystals and of their chains, can, under certain environmental conditions, persist for a geologically long time. The stability of the magnetization of magnetite crystals in chains is primarily controlled by magnetostatic interaction which ensures that even grains with a size of about 30 nm have their magnetic moments in the blocked state [15,16]. The advantages of the physicochemical and biological properties of bacterial magnetosomes relative to synthetic magnetic nanoparticles are giving rise to growing interest from medical science [17].

Magnetic properties of biogenic magnetite from magnetofossils are studied using standard paleo- and rock magnetism methods such as alternating fields or, in some cases, thermal demagnetization, and measurement of hysteresis loops, isothermal remanent magnetization acquisition curves, and saturation isothermal remanent magnetization (SIRM) demagnetization by dc magnetic field of the opposite sign (backfield curves). More advanced approaches include construction of first-order reversal curve diagrams [18,19], SIRM measurements at cryogenic temperatures [20], and ferromagnetic resonance (FMR) spectroscopy [21]. The influence of the external environment leads to a change in the chemical composition and structure of biogenic magnetite: Stable under slightly alkaline conditions of sea bottom sediments [22], it is partially or completely converted to maghemite during oxidation in freshwater conditions [6]. In the case of an increase in the oxygen content, coercive force of the particles tends to decrease [8]. The acidic environment determines the predominantly isotropic shape of magnetite crystals [23]. At an ambient temperature, coercivity of remanence *H_cr_* is between 10–50 mT, and the ratio of remanent saturation magnetization to saturation magnetization *M_rs_*/*M_s_* is 0.4–0.5 for most of described types of MTB [24,25,26,27,28].

A comparative study of the magnetic properties of whole MTB cells and isolated magnetosomes revealed their significant difference. In particular, for *Magnetospirillum magneticum* AMB-1 [29], the ratios *H_cr_*/*H_c_* = 1.25 and *M_rs_*/*M_s_* = 0.46 for the whole cell sample and *H_cr_*/*H_c_* = 1.5 and *M_rs_*/*M_s_* = 0.33 for the isolated magnetosome sample. The difference may be due to the predominance of uniaxial single-domain behavior in the case of the whole cell sample and increasing contribution of superparamagnetic behavior in the case of the isolated magnetosome sample. Resonance curves obtained by FMR spectroscopy differ for intact MTB culture, surface sediments, and magnetofossils located at various depth, and also depend on the asymmetry ratio of magnetite crystals [30]. The temperature of the Verwey transition characteristic of magnetite [31] in the case of MTB ranged between 90–120 K [24,25,26,32,33,34], with lower values corresponding to more oxidized varieties [24].

In this study, we described for the first time the morphology, crystallography, and magnetic properties of bacterial magnetosomes produced by *Magnetospirillum caucaseum* SO-1, one of the high-productivity MTB strains that is promising for future biomedical applications.

## 2. Materials and Methods

### 2.1. Bacterial Strain and Culture Conditions

Lyophilized non-pathogenic magnetotactic bacteria *Magnetospirillum caucaseum* SO-1 was obtained from the collection of Institute of Bioengineering, Research Center of Biotechnology of the Russian Academy of Sciences, Moscow. The strain was firstly isolated from a sediment sample from the Ol’khovka River, Kislovodsk, Caucasus, Russia [35]. The medium included (per liter of medium): 0.7 g of KH_2_PO_4_, 0.5 g of sodium succinate, 0.1 g of yeast extract, 0.35 g of NaNO_3_, 10 mL of 0.01 M ferric citrate, and 0.05 g of sodium thioglycolate. pH constituted 6.75. The bacteria were cultivated at 28 °C under microaerobic conditions (~1% oxygen in nitrogen) in a 15-L fermenter for 3–4 days. As reported previously, *Magnetospirillum caucaseum* SO-1 could not grow aerobically in the absence of a reducing agent, in contrast with one of the most frequently cultivated strain *Magnetospirillum magneticum* AMB-1 [35]. The strain SO-1 is characterized with wide growth temperature range of 18–42 °C with the optimum at 28 °C. In addition, it demonstrates high tolerance to oxygen (up to 21%) and ability to grow at various substrates including tartrate, propionate, butyrate, and glycerol [35]. Besides the potential biotechnological application of strain SO-1, its magnetofossils can be considered as potential carriers of paleomagnetic and/or paleoenvironmental signals in the geographical area of their habitat, North Caucasus, Russia.

Lyophilization of MTB and isolation of bacterial magnetosomes were obtained according to the protocol described in [36]. After achieving growth stationary phase *Magnetospirillum caucaseum* SO-1 cells were centrifuged 10,000× *g* for 10 min at +4 °C, resuspended in 20 mM of HEPES buffer, pH 7.4, containing 4 mM of EDTA and 0.1 mM of phenylmethylsulfonyl fluoride (PMSF) and disrupted by sonication (Sonopuls, Bandelin, Germany). Magnetosomes were isolated from disrupted cell fractions using a neodymium–iron–boron (Nd-Fe-B) magnetic stand and washed 15 times with 20 mM of HEPES buffer, pH 7.4. Finally, magnetosomes were resuspended in the same buffer and stored at +4 °C. The portion of purified magnetosomes was dried at 105 °C and weighted, thus evaluating the concentration of the remaining portion.

For measurements, the powder of isolated magnetosomes with a mass of 5.1 mg was divided into 5 parts in order to carry out, in parallel, studies of shape and size, chemical composition, crystal structure, and magnetic properties, as well as to avoid the effect of magnetite oxidation (maghemitization) on the experimental results.

Additionally, lyophilizate of MTB cell fragments was used for electron microscopy analysis to visualize cells form and to prove the presence of magnetosomes chains in the cell fragments in order to explain the magnetic properties.

### 2.2. Physico-Chemical Characterization of Magnetosomes and Cell Fragments

#### 2.2.1. X-ray Diffraction

Crystal structure analysis of isolated bacterial magnetosomes has been performed by X-ray diffraction (XRD) using a D2 Phaser diffractometer (Bruker, Billerica, MA, USA). One milligram of magnetosomes was mixed with petrolatum and then used for the analysis. The obtained XRD patterns were processed using the PDXL-2 software package (Rigaku, Tokyo, Japan) with the PDF-2 XRD database (International Center for Diffraction Data, 2011).

#### 2.2.2. Raman Spectroscopy

Phase composition of the isolated magnetosomes was additionally studied by Raman spectroscopy using a LabRam HR800 instrument (Horiba Jobin-Yvon, Kyoto, Japan). The second harmonic of a Nd:YAG laser (excitation wavelength 532 nm) was used as an excitation source. Laser radiation was focused on the sample surface into a spot with a diameter of ~1–2 μm. The same amount of the sample was used further for the transmission electron microscopy studies, since possible thermal destruction was negligible and could not influence the shape and size of magnetosomes.

#### 2.2.3. Electron Microscopy

The shape and size of the isolated magnetosomes and of the MTB cells fragments were studied by scanning electron (SEM) and transmission electron (TEM) microscopies using Quanta Inspect F50 (FEI Company, Eindhoven, The Netherlands) and JEM-2100F (JEOL, Tokyo, Japan) electron microscopes, respectively. In addition, X-ray energy dispersive analysis (EDX) of the elemental composition of the sample and visualization of the internal structure of lyophilized MTB by high-angle annular dark-field scanning transmission electron microscopy (HAADF STEM) were carried out using a JEM-2100F microscope. The sample for SEM studies was fixed using graphite-conductive double-sided tape and distributed evenly by non-magnetic tweezers. Samples for TEM studies were prepared by depositing the material on a standard copper TEM grid with an ultra-thin amorphous carbon backing film.

#### 2.2.4. Dynamic Light Scattering

The particle size distribution of the isolated magnetosomes was assessed by dynamic light scattering (DLS) using original measuring equipment developed at Peter the Great St. Petersburg Polytechnic University [37]. For measurements, a sample of 1 mg of isolated magnetosomes was resuspended in distilled water. Large aggregates fraction was separated using sedimentation during 720 h. The magnetosomes suspension was additionally homogenized just before measurements using ultrasonic bath Codyson CD-6800 (Shenzhen Codyson Electrical Co., Ltd., Shenzhen, China) for 10 min at ultrasonic power 50 W and frequency 42 kHz.

#### 2.2.5. Static Hysteresis and Thermal Magnetic Properties

The sample for magnetic measurements was the powder of isolated magnetosomes in amount of 1 mg pressed into a polymeric cylindrical sample holder with an outer diameter of 3 mm and a length of 6 mm. The paramagnetic signal was subtracted when constructing the hysteresis curve. Saturation isothermal remanent magnetizations (SIRM) acquired in a 5-T field at 5 K after zero field cooling (ZFC) and cooling in a strong (5 T) field (FC), respectively, were traced during the subsequent warming to 300 K in a zero field. SIRM acquired in a 5-T field at 300 K was measured during the zero-field cooling-warming cycle between 300 and 5 K. These experiments were carried out using a Quantum Design (US) MPMS 3 instrument in the vibration sample magnetometer (VSM) mode, with temperature sweeping at 2 K/min. A (near-) zero field to acquire the remanent magnetization was produced by setting the 2.7-mT nominal field in the MPMS “No overshot’ mode. Measurements of the Pd standard sample at 298 K showed that this procedure generally yields a residual field below 10 µT. The magnetic hysteresis loop in a 7-T maximum field and the backfield curve of SIRM acquired in a 5-T field were measured at 295 K using the same instrument.

#### 2.2.6. Electron Paramagnetic Resonance Spectroscopy

The electron paramagnetic resonance (EPR) of the samples was analyzed on a spectrometer Bruker EPR ELEXSYS E580 (X-BAND). The measurements were carried out in the standard mode with a modulation frequency of 100.00 kHz and a modulation amplitude of 0.5 mT at a 15.00 mW microwave power: The measurement time was 600 s and the temperature was 295 ± 1 K. Magnetic field was swept at a 0.166 mT/s rate. If necessary, the spectrum was accumulated. Spectra were processed using the Xepr and Origin 9 software package. Regardless of the signal-to-noise ratio, at the first stage, an EPR spectrum was smoothed using Savitsky–Golay algorithm [38].

#### 2.2.7. Theoretical Modeling

Theoretical analysis of the magnetic properties was carried out using the model of clusters of magnetostatically interacting two-phase particles [39]. Theoretical values of saturation magnetization and remanent saturation magnetization were calculated using a modified method of moments for a system of dipole–dipole-interacting uniaxial particles, as in [40,41].

## 3. Results and Discussion

### 3.1. Morphology and Chemical Composition

**Figure 1:**
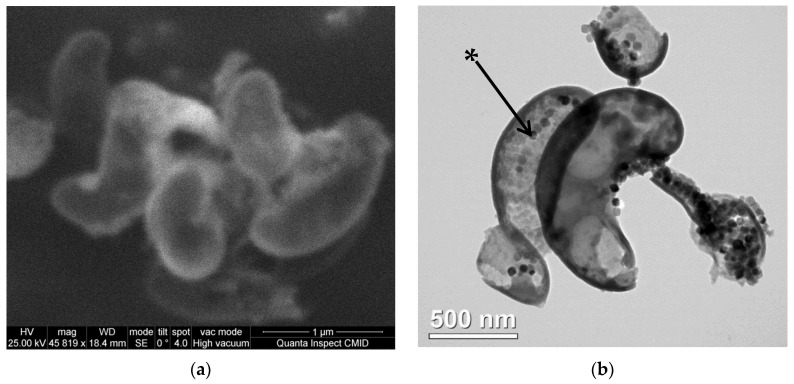
Electron microscopy images of lyophilized cell fragments of magnetotactic bacteria *Magnetospirillum caucaseum* SO-1: (**a**) Scanning electron microscopy image; (**b**) transmission electron microscopy image. The magnetosome in a cell fragment is marked with an asterisk.

SEM and TEM images of the lyophilized *Magnetospirillum caucaseum* SO-1 are shown in Figure 1.

As it can be seen from the images, lyophilized MTB cell fragments have the length of about 0.7–1.4 µm and a width of about 0.2–0.5 µm. The results are in general correlation with the previously reported [35], 0.3-µm-wide and 1.2–3.0-µm-long spiral-shaped motile, bipolarly flagellated cells. Magnetosomes form chains, characteristic of spirilla of the genus *Magnetospirillum* [42], which can be seen in lyophilized MTB. MTB cell fragments contain single chains of magnetosomes in accordance with the literature data [35] and also aggregates of magnetosomes not organized in chains.

**Figure 2:**
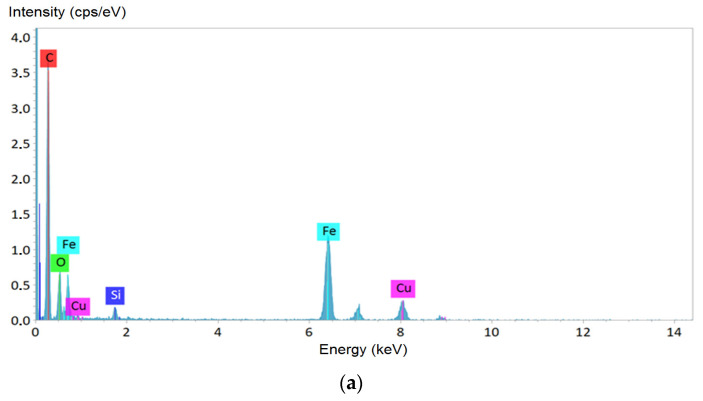

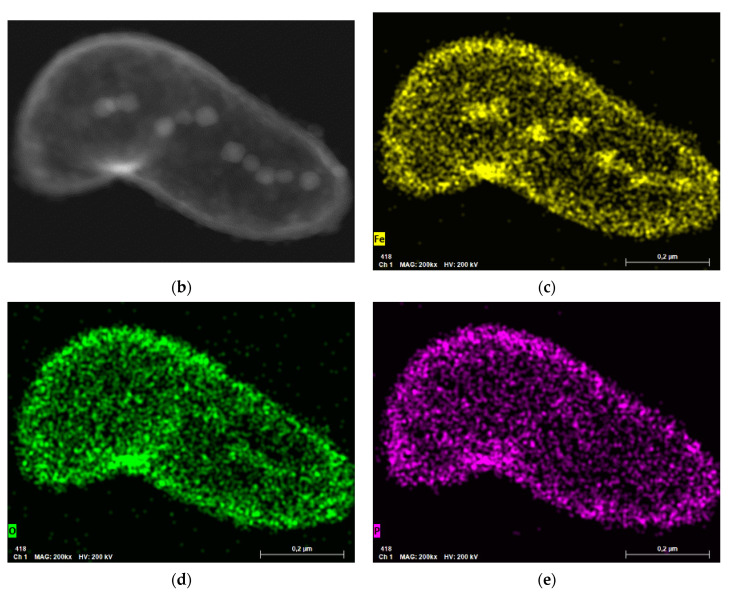
Analysis of the elemental composition of magnetotactic bacteria (MTB) lyophilizate: (**a**) X-ray energy dispersive analysis results; (**b**) internal structure of MTB cell fragment; (**c**–**e**) X-ray Fe, O, and P mapping of an MTB cell, respectively.

Results of the analysis of the elemental composition of the lyophilized MTB by EDX method, and X-ray maps of iron distribution obtained by STEM are presented in Figure 2.

Elemental composition of the MTB lyophilizate confirmed that the observed crystal grains were iron-containing inclusions, and not, e.g., granules of phosphates or hydroxides, also formed by MTB [43], since there was no evidence of P in the X-ray map. The copper peaks observed in the spectra arose due to a relatively massive copper support grid of the TEM sample. Silicon peaks may be the contribution of the supporting film of the TEM sample, but there was no reliable explanation within the framework of the current study, and therefore it requires further investigations to confirm or to exclude the presence of this element in the form of amorphous silica or other chemical compounds in the MTB lyophilizate.

**Figure 3:**
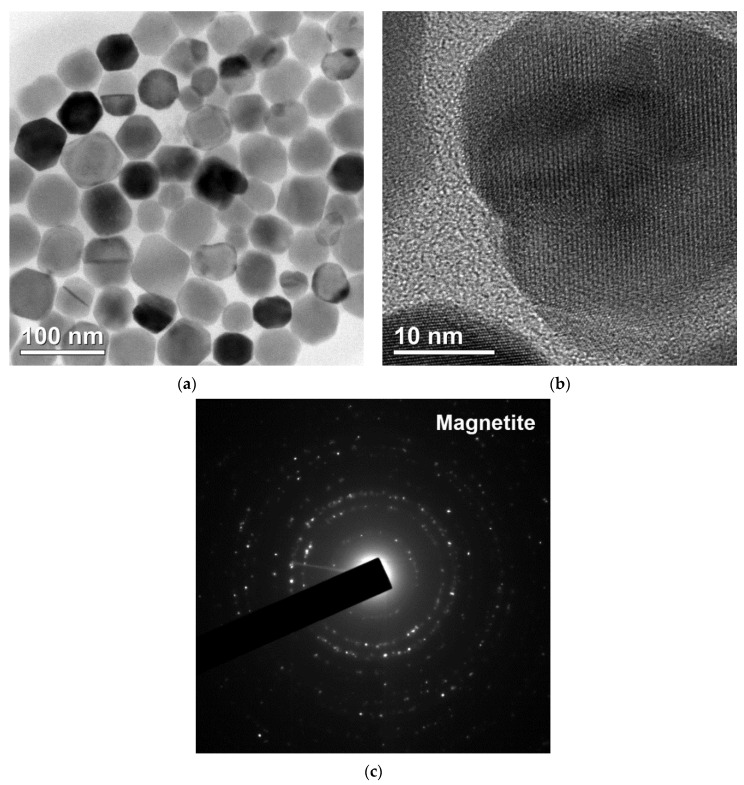
Transmission electron microscopy images of the isolated magnetosomes: (**a**) crystals of magnetosomes without membrane, (**b**) crystal lattice of an individual magnetite grain, (**c**) electron diffraction pattern.

The results of TEM analysis of the isolated magnetosomes are shown in Figure 3.

The shape of the isolated magnetosomes was close to cube-octahedral, and the characteristic sizes were 40–60 nm, in agreement with the previously obtained data [44,45,46].

**Figure 4:**
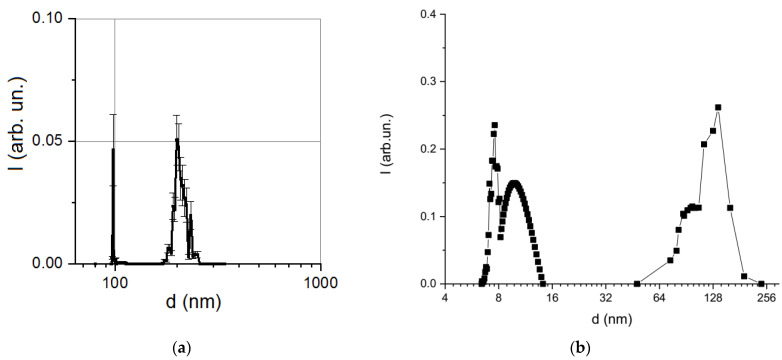
Comparison of particle size distribution obtained by the dynamic light scattering method: (**a**) the isolated bacterial magnetosomes; (**b**) synthetic magnetite-silica nanoparticles (reprinted from [37], Velichko, E.; Nepomnyashchaya, E.K.; Gareev, K.G.; Martínez, J.; Maicas, M.C. Characterization of Magnetite–Silica Magnetic Fluids by Laser Scattering. *Appl. Sci.* **2021**, 11, 183, doi:10.3390/app11010183, license CC BY 4.0).

The presence of both isolated magnetosomes and their aggregates resulted in a multimodal particle size distribution obtained by the DLS method (Figure 4). A local intensity maximum at about 100 nm corresponded to an average hydrodynamic diameter of individual magnetosomes. The next maximum at 200 nm may indicate the formation of aggregates of several isolated magnetosomes due to disruption of membrane integrity, since under the condition of membrane integrity magnetosomes isolated from MTB were characterized by high aggregate stability [47]. Particle size distribution, obtained for synthetic magnetite-silica nanoparticles using the same DLS technique, is shown in Figure 4b.

As shown in Figure 4b, the size distribution of the synthetic nanoparticles was also bimodal and was much wider due to the aggregation processes provoked by dilution [37]; therefore, as compared to the studied bacterial magnetosomes, such synthetic nanoparticles were not suitable for biomedical use.

**Figure 5:**
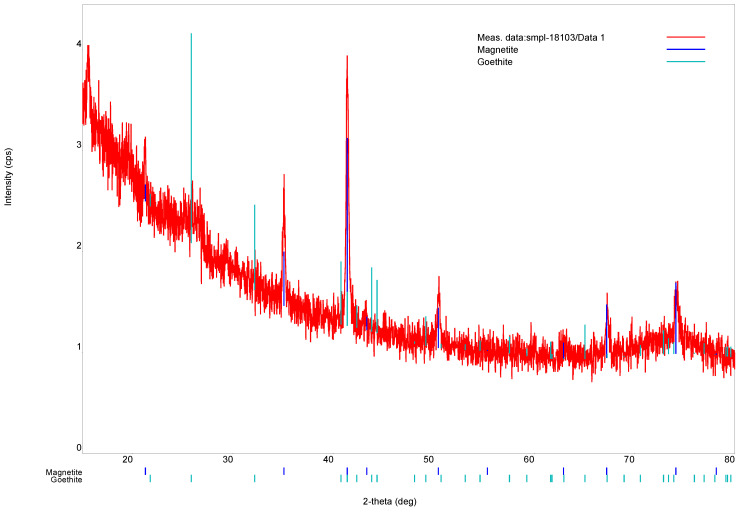
X-ray diffraction pattern of the isolated magnetosomes.

**Figure 6:**
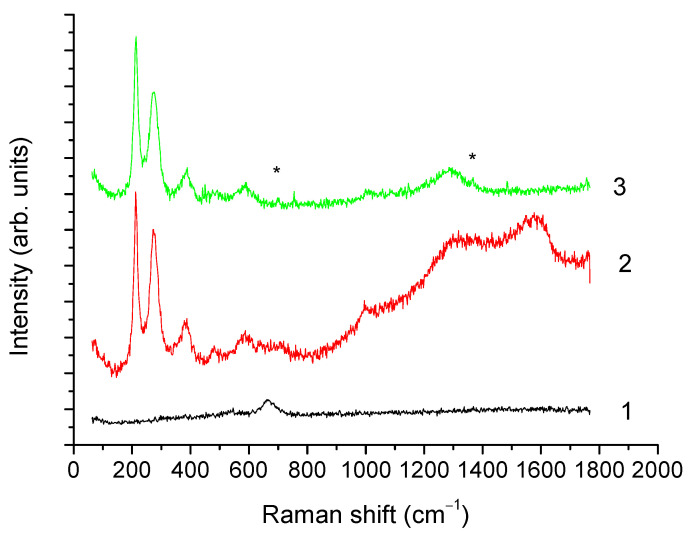
Raman spectra of the isolated magnetosomes; obtained at laser power 0.08 (curve 1), 0.8 (curve 2), and 2 mW (curve 3). * The 700 and 1370 cm^−1^ bands.

XRD and Raman spectroscopy were used to confirm the crystal structure and chemical composition of bacterial magnetite grains. The results are shown in Figure 5 and Figure 6, respectively. Crystals appeared to consist of magnetite (98%) and minor goethite (2%). The convergence factor of the calculated and experimental X-ray profiles is as follows:

(1) $$R_{P}=\frac{\Sigma \left|y_{i}^{obs}-y_{i}^{calc}\right|}{\sum y_{i}^{obs}}=6.2\%$$

where *y_i_* is the intensity at each experimental point of the X-ray diffraction pattern. The lattice parameter of magnetite was *a* = 0.8388 nm, which was slightly lower than the value for the stoichiometric composition (0.8398 nm, [48]) and approximately corresponded to the formula Fe_2.95_O_4.05_ [49].

XRD results are confirmed by the Raman spectroscopy data (Figure 6). At the minimum laser power (curve 1, laser power 0.08 mW), a single band at 670 cm^−1^ was observed, attributed to *A*_1*g*_ vibrations of magnetite [50]. Under the action of 0.8 mW power laser radiation (curve 2), annealing of the sample was observed, leading to the appearance of narrow lines in the range from 200 to 500 cm^−1^ and of broad bands between 1200 and 1650 cm^−1^. Presence of narrow lines with maxima near 220, 247, 412, and 498 cm^−1^ indicated the recrystallization of magnetite into hematite. The appearance of broad bands in the range from 1200 to 1650 cm^−1^, characteristic of various forms of carbon, may be related to the presence of organic matter. Further increase of the laser radiation power to 2 mW carried out for laser annealing of the sample (curve 3) similar to [50], led to a decrease in the intensity of the 1200 to 1650 cm^−1^ bands, likely caused by a partial removal of organic matter of magnetosomes membrane, whereas the lines characteristic of hematite remained unchanged.

It is worth noting that the observed shift of hematite lines towards lower frequencies relative to the bulk material [51] is characteristic of nanosized iron oxide particles [50,52]. In addition, in the spectra after laser annealing, narrow low-intensity lines near 700 and 1370 cm^−1^ (marked with asterisks) were observed, which may indicate the coexistence of maghemite (characteristic lines 700, 1370, 1560 cm^−1^) and hematite (lines 220, 247, 412, 498, 613, 1320 cm^−1^) phases.

Concordant results yielded by the above analytical methods indicate that the main volume of the isolated magnetosomes crystalline phase consisted of magnetite with grain size ranging from tens to the first hundreds of nm. Furthermore, despite the partial destruction of the magnetosome membrane, magnetite is characterized by a high chemical stability incomparable with respect to the chemical stability of synthetic nanoparticles subject to intense maghemitization [41].

### 3.2. Magnetic Properties

**Figure 7:**
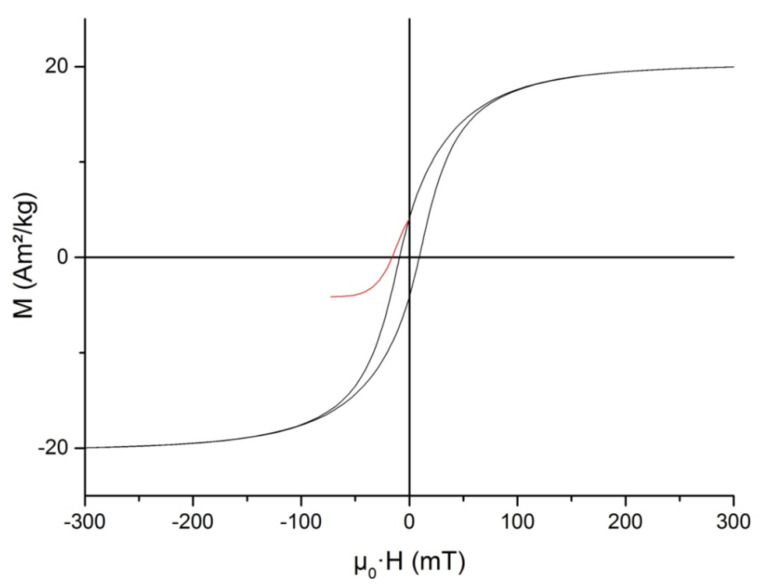
Central part of static hysteresis loop and saturation isothermal remanent magnetization backfield curve of the isolated magnetosomes measured at 295 K.

The static hysteresis loop and SIRM backfield curve measured at 295 K are shown in Figure 7. The hysteresis parameters were as follows: saturation magnetization was *M_s_* = 20.46 Am^2^/kg; saturation remanent magnetization *M_rs_* = 4.11 Am^2^/kg; their ratio (squareness) *M_rs_*/*M_s_* = 0.201; coercive force μ_0_*H_c_* = 9.09 mT; remanent coercivity μ_0_*H_cr_* = 16.03 mT; and ratio *H_cr_*/*H_c_* = 1.76. The value of saturation magnetization, *M_s_*, was about 20% of the saturation magnetization of magnetite or about 26% of that of maghemite [53]. *H_c_* and *H_cr_* values lay in the range reported in the literature. On the other hand, the *M_rs_*/*M_s_* ratio was approximately two times lower than for magnetosomes isolated from cells of other representatives of the genus *Magnetospirillum* (Table 1) and was closer to that for synthetic interacting single-domain magnetite particles [54]. The main reason for this discrepancy seemed to be that in our samples, magnetosome chains were mostly dismembered and, in fact, we were dealing with nearly isometric clusters of magnetite particles (*cf.* Figure 3a).

**Figure 8:**
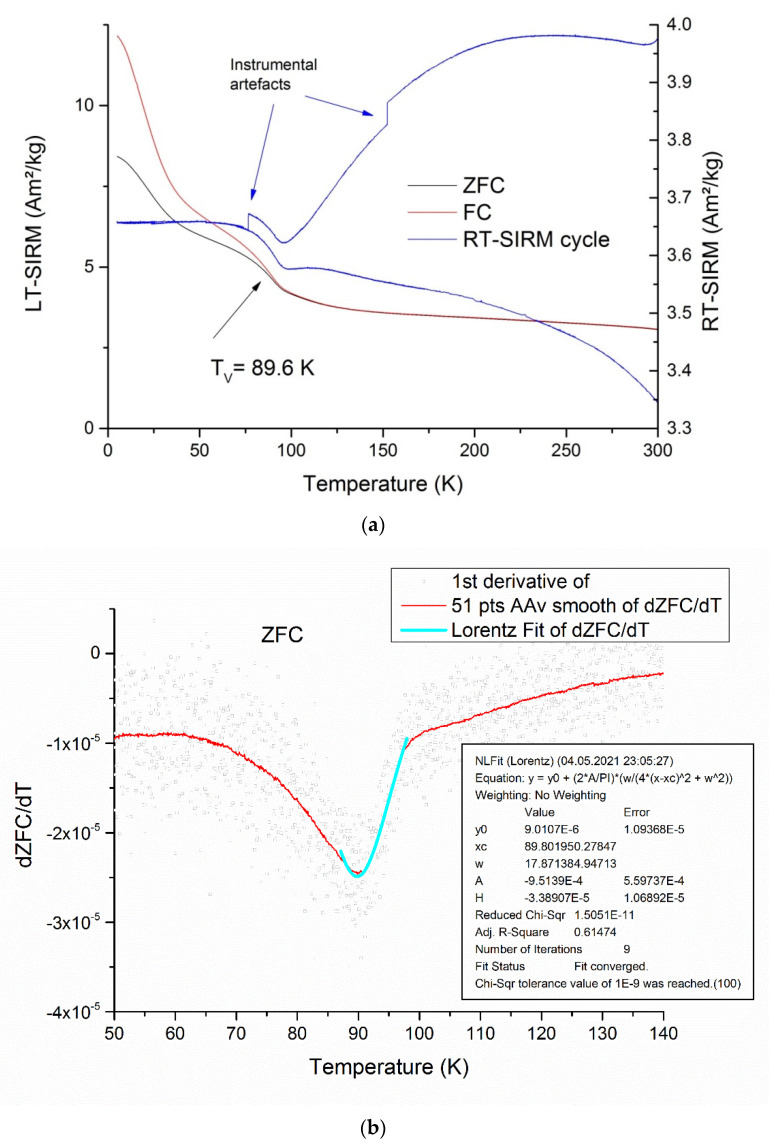
Thermal magnetic properties of the isolated magnetosomes: (**a**) zero field cooling (ZFC) and cooling in a strong (5 T) field remanence curves (left ordinate axis) and zero-field cycling of saturation isothermal remanent magnetization given at 300 K; (**b**) determining the Verwey transition temperature by Lorentz approximation of ZFC/dT temperature dependence.

Another reason for a decrease of the *M_rs_*/*M_s_* ratio may be partial oxidation of magnetite grains, which manifested itself in a significant, down to 89.8 K, decrease in the Verwey transition temperature (Figure 8a) compared to the 125 K characteristic of stoichiometric magnetite [59].

A relatively fast decrease of SIRM given at a low (say, 10 K and below) temperature with a gentle break-in-slope at 40–50 K is a hallmark of nonstoichiometric magnetite, both of inorganic [53,60,61] and of biogenic origin [33,62,63]. A *T_v_* value of about 90 K corresponded to Fe_3(1–__δ)_O_4_ compositions with δ = 0.008–0.012 [64,65], which generally agreed with that inferred from the XRD data. It is worth noting that *M_rs_*/*M_s_* values of the order of 0.2, which are not typical for magnetosome chains, are known to occur for nonstoichiometric magnetite with a grain size of about 40 nm [53,60].

**Figure 9:**
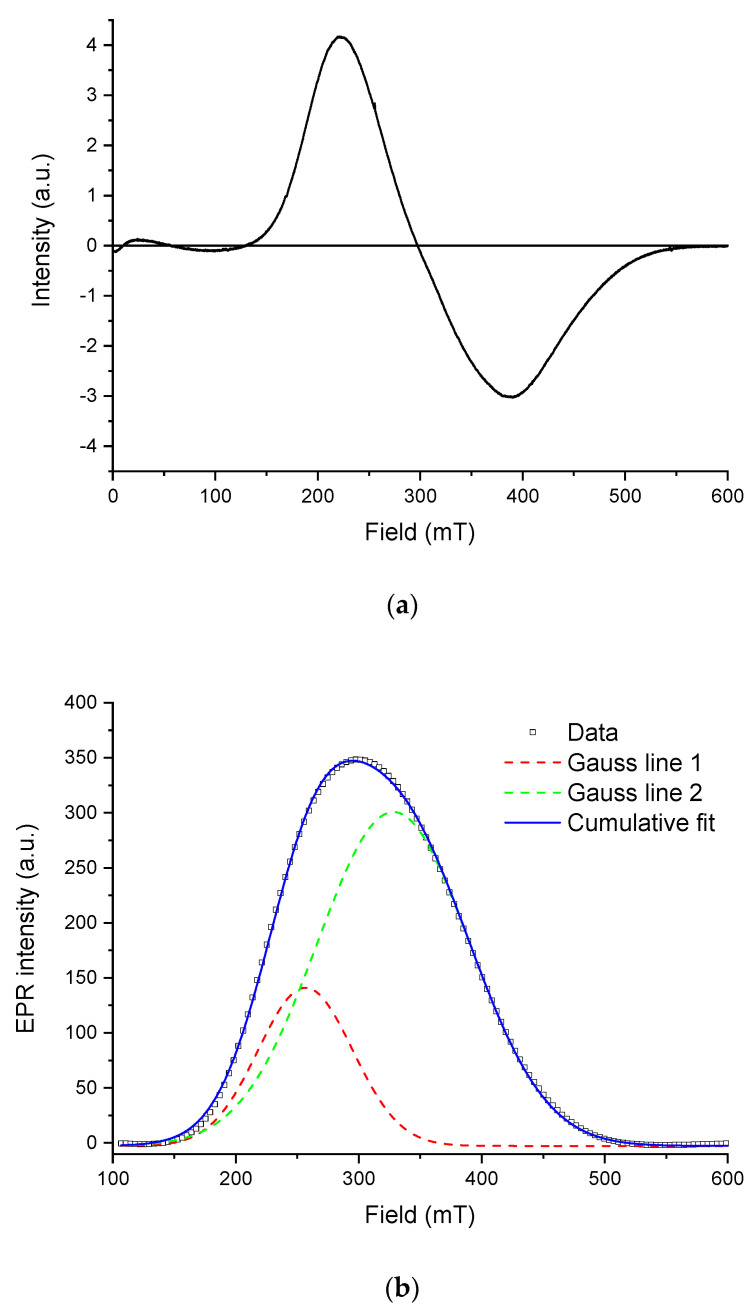
Electron paramagnetic resonance (EPR) spectroscopy of the isolated magnetosomes: (**a**) EPR spectrum measured at room temperature; (**b**) decomposition of the first derivative (solid line) of the EPR signal (absorption spectrum) into two Gaussian components (colored dashed lines). Bold line shows the cumulative fit.

Figure 9 shows the EPR spectrum of the isolated magnetosomes (a) and its approximation with two individual bands using the Gaussian function (b).

The spectrum is an asymmetric inhomogeneously broadened line (∆*B_pp_*~167 mT), with the shape characteristic for EPR spectra of Fe^3+^ cations coupled by a strong exchange interaction between ions in different charge states [66]. This shape of the resonance curve is commonly observed in magnetite from fossilized MTB [21,67,68], where the grains have a lognormal size distribution and are arranged mainly in chaotic aggregates rather than in ordered chains [69], in agreement with electron microscopy observations. The spectrum can be decomposed into two symmetric Gaussian components with *g*-factors of 2.01 and 2.32, respectively. This may be viewed as evidence for the presence of two types of particles and/or their aggregates in the sample. In this line, it is noteworthy that oxidation of magnetite in magnetofossils is expressed in the EPR spectra by a significant increase of the effective *g*-factor, which is close to 2.00 for stoichiometric magnetite [28,68,70].

### 3.3. Theoretical Modeling

In accordance with DLS data (Figure 4), for further modeling of the magnetic characteristics, aggregates about 100–200 nm in size, containing 1 or 2 isolated magnetosomes which correspond to both maxima in Figure 4a, were assumed.

The characteristic size of individual grains in both aggregates (*cf.* Figure 3) was assumed to be 40 nm. For an aggregate of the first type, in which approximately 10 grains were arranged in a chain, the size of the cluster (cylinder) corresponded to the size of the bacterium (Figure 1) with the height of about 750 nm and the diameter of about 300 nm. For an aggregate of the second type, in which we assume a uniform distribution of grains of the same size (40 nm), the height and diameter of the cluster were chosen to be about 6 µm. From Figure 4, the relative volumetric fractions of two types of aggregates (clusters) were estimated to be 0.06 and 0.94, respectively.

The theoretical value of the magnetization reversal field (microcoercivity) of an isometric single-domain magnetite grain $H_{0}=\frac{4}{3}\cdot \frac{K_{u}}{I_{s}}$, where $K_{u}\approx \left|K_{1}\right|=1.35\cdot 10^{4}$ J/m^3^ is the constant of crystallographic anisotropy and $I_{s}\approx 471$ kA/m is the spontaneous saturation magnetization [71]. This corresponds to μ_0_*H*_0_ = 37.5 mT. Experimentally, average microcoercivity was expected to be close to a measured value of remanent coercivity. Our experimental value of the latter was μ_0_*H_cr_* = 16.0 mT. In the case of an aggregate of the first type, remanent coercivity *H_cr_* can be estimated using the Jacobs–Bean model [72] for a chain of 10 magnetic spheres as follows:

(1). With parallel rotation of the magnetic moments of the spheres (μ_0_*H_c_*_1_ = 62.9 mT);
(2). With fan-shaped magnetization reversal (μ_0_*H_c_*_2_ = 25.3 mT).

The size of single-domain magnetite of spherical shape is 29–36 nm [54]. In our case, grains have not spherical but parallelepipedal form and their characteristic size exceeds 40 nm. It should be noted that the presence of goethite according to the XRD data (see Figure 5) and the fact that the characteristic grain size exceeds the size of single-domain magnetite leads to nonuniform distribution of the magnetic moment in the grain. This leads to an underestimation of the spontaneous saturation magnetization *I_s_* of the grain. Taking into consideration the chemical and magnetic inhomogeneity of distribution of the magnetic moment in the grain and the relative contribution of the two types of aggregates to the magnetic parameters, based on the model of magnetostatically interacting two-phase particles [40,41], measurements of static hysteresis loops consistent with the experimental data were carried out (see Paragraph 3.2 and Figure 7) theoretical modeling of these parameters (*M_s_*, *M_rs_*, *H_c_ H_cr_*).

If we assume that the effective (average) spontaneous magnetization *I_eff_* of a two-phase “magnetite/goethite” grain in a ratio of 0.98/0.02 at saturation is about 450 kA/m, then the best agreement with experiment is achieved at the following values of the parameters:

(1). By remanent magnetization *M_rs_* − *I_eff_* (chain) = 380 kA/m, *I_eff_* (group) by *M_rs_* = 280 kA/m and average over the sample *I_eff_* (theory) = 286 kA/m;
(2). In coercivity *H_cr_* − μ_0_*H*_0_(chain) = 41 mT, μ_0_*H*_0_(group) = 15 mT, and average over the sample μ_0_*H*_cr_ (theory) = 16.5 mT.

Therefore, it can be assumed that magnetostatically interacting grains in the isolated bacterial magnetosomes are chemically and magnetically inhomogeneous, which leads to a decrease in magnetization and average coercivity of the sample.

## 4. Conclusions

We experimentally for the first time studied the morphology, chemical composition, and magnetic properties of magnetosomes isolated from magnetotactic bacterium *Magnetospirillum caucaseum* SO-1, a high-productivity MTB strain which is promising for various biomedical applications. Magnetofossils of SO-1 can be considered as potential carriers of paleomagnetic and/or paleoenvironmental signals in the local geographical area of their habitat. Despite the absence of special conditions such as an inert atmosphere and controlled storage medium pH, the bacterial magnetosomes demonstrated very high aggregate and chemical stability and changed only slightly compared to the reference one for this MTB species and compared to the synthetic iron oxide nanoparticles.

Bacterial magnetite grains apparently become considerably oxidized at the surface, as indicated by the presence of goethite, while the grain volume develops a weaker (the volume of the crystal becomes smaller), of the order of δ = 0.008–0.012 in the Fe_3(1–δ)_O_4_ formula, a deviation from stoichiometry which manifests itself in a decrease in the Verwey transition temperature to 89.8 K. Magnetic properties of the sample appeared to be controlled mostly by random aggregates of magnetosomes, with a minor contribution from preserved magnetosome chains, and showed a striking contrast with the properties of isolated, non-interacting single-domain particles of magnetite. Chemical and, by inference, magnetic properties of magnetofossils were thus expected to be highly stable compared to abiogenic magnetite particles.

## Figures and Tables

**Table 1:** Comparison of the hysteresis characteristics of bacterial magnetosomes isolated from various species (strains) of MTB at room temperature.

Species (Strain) of Magnetotactic Bacteria	*M_rs_*/*M_s_*	μ_0_*H_c_*, mT	μ_0_*H_cr_*, mT	*H_cr_*/*H_c_*	Refs.
*Magnetospirillum magneticum* AMB-1	0.31–0.51	3.7–24.7	–	–	[55]
	0.46	22.5	31.9	1.42	[18]
	0.46	32.0	38.0	1.19	[56]
	–	14.4	25.8	1.79	[34]
*Magnetospirillum gryphiswaldense* MSR-1	0.38	5.9	10.8	1.82	[27]
	0.44	16.3	20.3	1.25	[24]
	0.40	15.7	–	–	[28]
	0.45	16.8	–	–	[57]
	0.37	9.6	15.2	1.58	[26]
	0.45	22.0	–	–	[58]
*Magnetospirillum caucaseum* SO-1	0.20	9.1	16.0	1.76	This work

## Data Availability

Not applicable.
